# Endoscopic ultrasound-guided portal vein marking facilitates transjugular intrahepatic portosystemic shunt for portal cavernoma

**DOI:** 10.1055/a-2742-5938

**Published:** 2025-12-08

**Authors:** Jun Li, Yingqun Zhou, Junshan Wang, Jiao Feng, Shilong Han, Min Yuan, Feng Liu

**Affiliations:** 1 278245 Department of Gastroenterology, Shanghai Tenth People’s Hospital, Tongji University School of Medicine, Shanghai, China; 2 278245 Department of Interventional and Vascular Surgery, Shanghai Tenth Peopleʼs Hospital, Tongji University School of Medicine, Shanghai, China

Portal cavernoma (PC), often resulting from chronic portal vein thrombosis in cirrhosis, poses a significant challenge for transjugular intrahepatic portosystemic shunt (TIPS) placement. The inability to visualize the occluded portal vein under fluoroscopy substantially increases procedural difficulty and failure rates 1 . Endoscopic ultrasound (EUS) has emerged as a potential tool to overcome this limitation by deploying a coil into the portal vein to enable direct portal vein targeting.

We present a case of a 68-year-old woman with HBV-related cirrhosis, previous splenectomy, recurrent variceal bleeding, refractory ascites and extensive PC ( Fig. 1 ). After multidisciplinary evaluation, a hybrid procedure was performed. Initially, in the endoscopy center, EUS-guided puncture of the main portal vein was successfully achieved via the duodenal bulb using a 22G needle and a 6 mm × 20 cm coil was deployed through the needle ( Fig. 2 and Video 1 ). The coil position was confirmed by immediate computed tomography ( Fig. 3 ). The patient was then transferred to the catheterization room. Using the coil as a target, the portal vein was successfully punctured from the hepatic vein in a single pass ( Fig. 4 ). Subsequent steps – guidewire advancement, pressure measurement, stent placement, and balloon dilation – were completed uneventfully ( Fig. 5 ). The patient recovered without procedure-related complications and was discharged after 2 weeks of medical treatments.

**Fig. 1:**
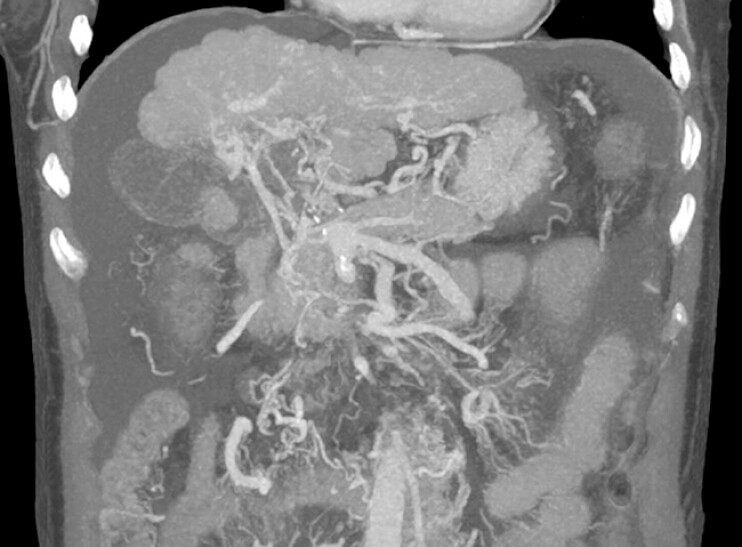
CT venography showed liver cirrhosis with PC and ascites. CT, computed tomography.

**Fig. 2:**
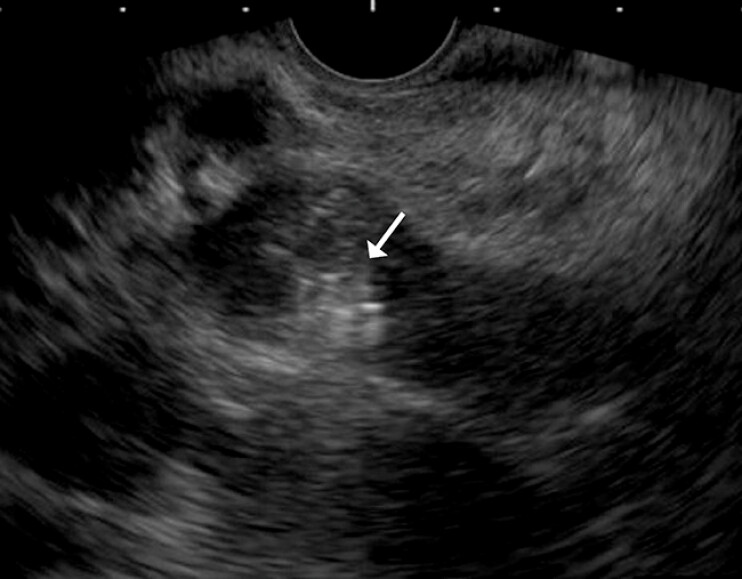
A 6 mm × 20 cm coil (white arrow) was deployed in the main portal vein.

EUS-guided portal vein marking facilitates transjugular intrahepatic portosystemic shunt for portal cavernoma. EUS, endoscopic ultrasound.Video 1

**Fig. 3:**
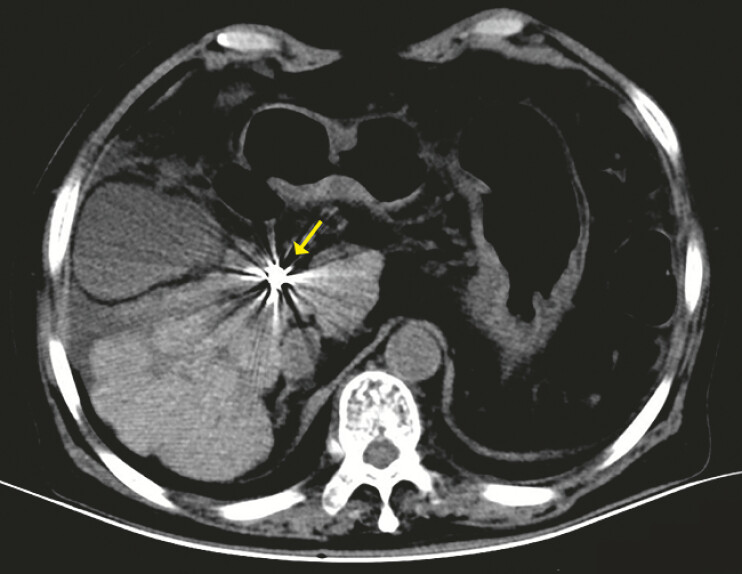
Immediate CT confirmed the coil position (yellow arrow). CT, computed tomography.

**Fig. 4:**
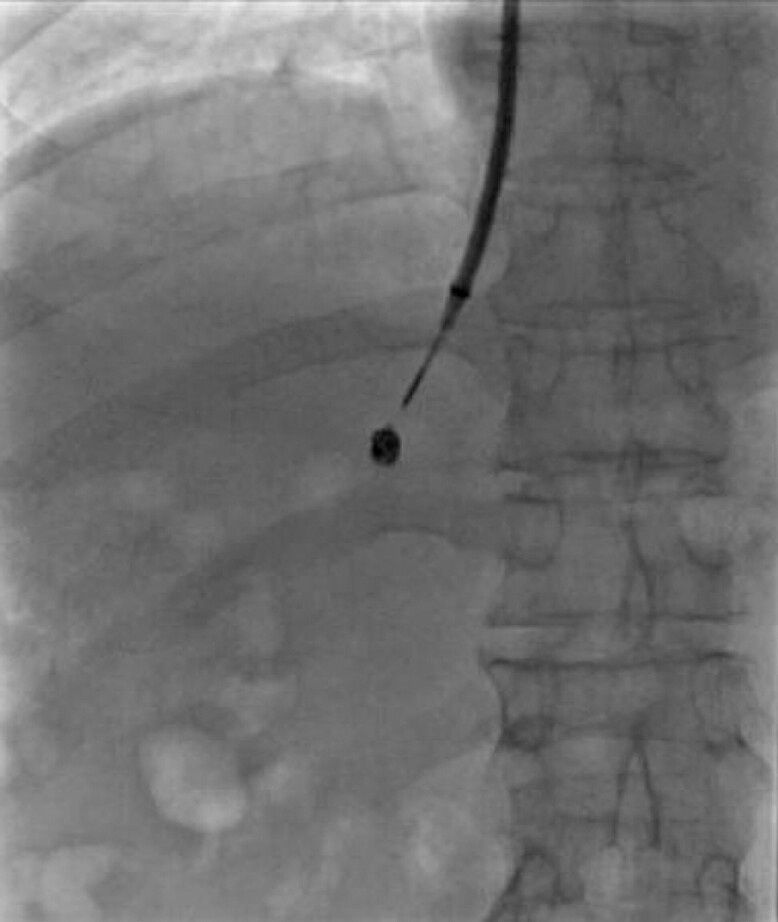
The portal vein was punctured from the hepatic vein using the coil as a target.

**Fig. 5:**
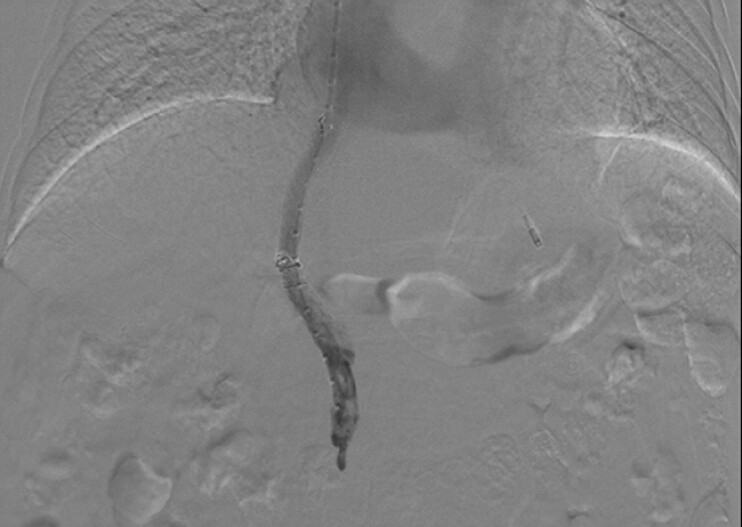
Angiography showed smooth flow of contrast through the stent into the inferior vena cava.

To our knowledge, this represents the first reported case of EUS-guided transduodenal portal vein marking for TIPS in patients with portal cavernoa. While a single Chinese report described a transgastric approach 2 , our transduodenal technique offers a shorter access route and avoids regions typically affected by esophagogastric varices. The risk of bleeding at the portal vein puncture site is very low in these patients due to portal vein occlusion and the following TIPS stent placement. This innovative approach enhances the feasibility and safety of TIPS in patients with PC.

Endoscopy_UCTN_Code_TTT_1AS_2AG

## Notes

Endoscopy E-Videos https://eref.thieme.de/e-videos
*E-Videos* is an open access online section of the journal *Endoscopy* , reporting on interesting cases and new techniques in gastroenterological endoscopy. All papers include a high-quality video and are published with a Creative Commons CC-BY license. Endoscopy E-Videos qualify for HINARI discounts and waivers and eligibility is automatically checked during the submission process. We grant 100% waivers to articles whose corresponding authors are based in Group A countries and 50% waivers to those who are based in Group B countries as classified by Research4Life (see: https://www.research4life.org/access/eligibility/ ). This section has its own submission website at https://mc.manuscriptcentral.com/e-videos .
